# Shifting resource dominance in career decision-making difficulties: a competitive mediation model of resilience and general self-efficacy among Chinese university students

**DOI:** 10.3389/fpsyg.2026.1768003

**Published:** 2026-03-17

**Authors:** Hongying Chang, Guohua Yan, Jichang Guo, Caifu Wu

**Affiliations:** Department of Psychology, School of Educational Sciences, Minzu Normal University of Xingyi, Xingyi, China

**Keywords:** career decision-making difficulties, Chinese university students, future work self clarity, general self-efficacy, multiple mediation, resilience

## Abstract

In contemporary labor markets characterized by heightened uncertainty and structural constraints, career decision-making difficulties (CDD) have become a salient psychological challenge for university students. Drawing on an integrated framework of future-oriented cognition and psychological resources, the present study examined how future work self clarity (FWSC) is associated with CDD through two distinct psychological resources—resilience and general self-efficacy (GSE)—and whether these resources operate in competing directions. A sample of 1,498 Chinese undergraduate students completed measures of FWSC, resilience, GSE, and CDD. Multiple mediation analyses were conducted using PROCESS Model 6 with bootstrapping procedures. Results showed that FWSC was directly and negatively associated with CDD. At the indirect level, FWSC exerted opposing effects on CDD through parallel mediators: resilience positively mediated the association between FWSC and CDD, whereas GSE served as a negative mediator. Due to the countervailing directions of these two indirect paths, the total indirect effect was nonsignificant, despite both specific indirect effects being statistically reliable. In addition, a significant serial indirect effect emerged, indicating that FWSC increased resilience, which in turn enhanced GSE and subsequently reduced CDD. These findings suggest that the influence of future-oriented cognition on career decision-making is not uniformly adaptive but depends on the configuration of psychological resources through which it is translated. Specifically, resilience may amplify decisional strain under conditions of high goal clarity and contextual constraint, whereas general self-efficacy provides a more stable protective pathway. By demonstrating a competitive multiple-mediation structure, this study highlights the differentiated functions of psychological resources in career decision processes and underscores the importance of considering resource configurations when addressing career decision-making difficulties among university students.

## 1 Introduction

Against the backdrop of profound global economic restructuring, accelerated technological transformation, and increasingly diversified—but increasingly constrained—career trajectories, university students are navigating career decision-making environments that are more complex and uncertain than ever before (Akkermans et al., 2020; Wang and Dong, 2024). Career choice is no longer a one-off, linear event but has evolved into a dynamic and career-constructive process, in which decisions are continuously shaped and reshaped over time across changing life and labor-market contexts (Savickas, 2013; Lent and Brown, 2020, Xu, 2023). At any stage of this process, heightened environmental uncertainty, cognitive ambiguity, or insufficient psychological resources may undermine individuals’ decisional capacity, giving rise to substantial career decision-making difficulties (CDD), characterized by confusion, hesitation, and decisional paralysis (Gati et al., 1996; Levin et al., 2024; Arbona et al., 2024).

A growing body of research indicates that CDD constitutes a critical psychological barrier during the school-to-work transition, exerting far-reaching effects on employment quality, psychological well-being, career development trajectories, and the formation of vocational self-concepts (Priyashantha, 2023; Arbona et al., 2024). Within the Chinese context, characterized by intensified labor market competition and pronounced structural constraints, CDD has become one of the most salient psychological challenges faced by university students during the career preparation phase. Beyond immediate employment outcomes, persistent decision difficulties may undermine long-term career adaptability and mental health (Priyashantha, 2023; Arbona et al., 2024; Ran et al., 2022).

Career decision-making difficulties refer to the confusion, hesitation, and decisional paralysis individuals experience during career choice processes due to cognitive, emotional, informational, or decisional-style factors (Levin et al., 2024). Conceptually, CDD has been widely understood as a multidimensional psychological condition encompassing lack of readiness, lack of information, and decisional conflicts (Gati et al., 1996). While early research primarily emphasized informational deficits, more recent studies have shifted attention toward the deeper psychological resource foundations of CDD. For instance, Xu (2024) demonstrated that deficiencies in psychological resources better explain core decision difficulties than objective information shortages. Similarly, a recent systematic review by Priyashantha, (2023) conceptualized CDD as a form of “resource vulnerability,” involving diminished future orientation, impaired emotion regulation, and low self-efficacy. Despite these advances, how multiple internal psychological resources jointly shape CDD—particularly through interactive or competing pathways—remains insufficiently examined.

One future-oriented construct that has received increasing scholarly attention is the future work self (FWS), introduced by Strauss et al. (2012), which refers to individuals’ mental representations of who they aspire to become in their future working lives. Within this framework, future work self clarity (FWSC) captures the degree to which this future work identity is cognitively clear, concrete, and stable. Importantly, FWSC emphasizes the accessibility and structural coherence of future-oriented self-representations rather than their moral value or evaluative significance, thereby distinguishing it from broader constructs such as purpose or calling (Strauss et al., 2012). A substantial body of evidence suggests that a clear future work self facilitates career exploration, career planning, and proactive career behaviors (Bernabé et al., 2023; Pignault et al., 2023; Yang et al., 2023). Cross-national research further demonstrates that FWSC robustly predicts career goal setting, planning behaviors, and exploratory tendencies among university students (Voigt and Strauss, 2024). Under conditions of heightened career uncertainty, FWSC has thus been conceptualized as a motivationally grounded, future-oriented psychological resource that helps individuals navigate career ambiguity and mobilize career-related actions (Raditya and Salim, 2025).

Nevertheless, the mechanisms through which FWSC influences career decision-making difficulties remain theoretically underdeveloped. Although prior studies generally report a negative association between FWSC and career uncertainty (Voigt and Strauss, 2024), they seldom unpack the internal psychological processes that may simultaneously amplify or attenuate this relationship. This issue is particularly salient in the Chinese graduate labor market, where strong future aspirations often coexist with structural constraints and limited opportunities. In such contexts, a clear future work self may not only guide action but also intensify perceived discrepancies between career goals and contextual feasibility, thereby producing differentiated psychological consequences through distinct resource pathways.

Resilience has traditionally been conceptualized as a recovery-oriented psychological resource that enables individuals to rebound from adversity and restore prior levels of functioning in the face of stress and uncertainty (Smith et al., 2008; Fletcher and Sarkar, 2013). In this sense, resilience primarily reflects individuals’ capacity to withstand and recover from stress, rather than an inherent tendency toward rigid persistence or inflexible goal pursuit. Although resilience has been widely associated with adaptive outcomes in educational and occupational contexts (Jeamjitvibool et al., 2022; Gong et al., 2023), emerging research cautions against assuming that its effects are uniformly protective across all decision-making situations.

In complex and high-stakes career contexts characterized by prolonged uncertainty and constrained opportunities, recovery-oriented resilience may indirectly sustain individuals’ cognitive and emotional engagement with challenging decision environments. Rather than facilitating timely disengagement or strategic reorientation, sustained tolerance of stress may extend exposure to decisional demands, thereby increasing cognitive load and ruminative processing (Troy et al., 2023; Liu et al., 2024). Importantly, this pathway does not suggest that resilience is equivalent to maladaptive persistence or rigid goal adherence. Instead, it highlights a more subtle mechanism through which resilience may maintain engagement under uncertainty, even when situational constraints limit feasible action options.

When future work self clarity (FWSC) activates a strong promotion-focused or ideal-oriented regulatory mode, individuals with higher levels of recovery-oriented resilience may remain cognitively and emotionally invested in career choices for extended periods. This perspective aligns with recent evidence emphasizing the nuanced roles that resilience plays as a psychological resource, showing that resilience interventions can differentially impact well-being and engagement (Abulfaraj et al., 2024), predict career decision self-efficacy and adaptability over time (Alkal, 2025), and correlate with sustained work engagement and job satisfaction outcomes (Ibrahim and Hussein, 2024). Moreover, resilience interacts with related constructs such as grit and self-efficacy to shape long-term adaptive processes in career contexts (Xu et al., 2025), and longitudinal evidence indicates that resilience supports broader well-being across individuals and organizations (Verma et al., 2025). Research on goal adjustment suggests that difficulties in timely disengagement from challenging or ambiguous goals can increase decisional strain, particularly in contexts where alternative pathways are unclear or structurally constrained (Wrosch et al., 2003). Under such conditions, resilience may be associated with higher levels of career decision-making difficulties—not because it promotes dysfunctional stubbornness, but because it sustains engagement in demanding decisional environments without necessarily facilitating adaptive disengagement or recalibration.

It should be acknowledged that recovery-oriented conceptualizations of resilience primarily capture stress tolerance and rebound capacity, rather than persistent or dysfunctional goal pursuit. Accordingly, the proposed mechanism represents an indirect and context-dependent pathway, and alternative psychological processes—such as overcommitment or maladaptive persistence—may also contribute to prolonged exposure to career decision demands. Clarifying these distinctions is essential for a more precise understanding of how resilience functions within complex career decision-making processes.

In contrast, self-efficacy occupies a central position within social cognitive career theory (SCCT) and has been consistently linked to career exploration, adaptability, and decision-making competence (Lent and Brown, 2020, Xu, 2023). Meta-analytic evidence confirms that self-efficacy serves as a critical protective resource in career decision-making, reducing uncertainty and enhancing decisional preparedness (Levin et al., 2024). Among university students, higher general self-efficacy (GSE) buffers career-related anxiety, strengthens career agency, and exerts enduring positive effects on career development (Zhou et al., 2024; Kwon (2019)). Given that FWSC enhances individuals’ perceived control over future possibilities, it is reasonable to expect that FWSC may reduce career decision difficulties by fostering stronger general self-efficacy beliefs.

Recent theoretical perspectives further emphasize that psychological resources rarely operate in isolation; instead, they influence career outcomes through multiple, and sometimes competing, pathways (Chan and Mai, 2021). Within the combined frameworks of psychological resource models and career construction theory (Savickas, 2013; Liu et al., 2023), FWSC may be conceptualized as a future-intentional resource, whereas resilience and self-efficacy represent stress-adaptation resources and action-efficacy resources, respectively. These resources differ not only in their content but also in their functional logic, regulatory focus, and behavioral consequences. For example, resilience may increase sustained exposure to challenge and uncertainty, whereas self-efficacy primarily reduces decisional anxiety and avoidance by facilitating action initiation and commitment. As a result, resilience and self-efficacy may form a competitive mediation structure, transmitting the effects of FWSC on career decision-making difficulties in opposing directions (Wang and Dong, 2024; Gürbüz et al., 2024).

Given the mixed implications of future-oriented self-concepts in career research, recent studies suggest that these constructs can exert both adaptive and strain-inducing effects, depending on resource configurations and contextual constraints. Future work self-clarity, for example, has been linked to proactive career behaviors and planning, though its effects may vary with individual resources and context (Söner and Yılmaz, 2025). Psychological resilience also plays a key role in career exploration and decision-making self-efficacy, interacting with these resources to influence how individuals cope with career uncertainty (Xu et al., 2025).

In contexts like contemporary Chinese labor markets, where competition is intense and opportunities are constrained, a clear future work self can both drive goal-directed action and highlight discrepancies between aspirations and feasible outcomes. As such, the present study hypothesizes that:

H1: Future work self clarity is significantly associated with career decision-making difficulties.

Resilience, often seen as sustaining engagement under prolonged uncertainty, may sometimes prolong cognitive load and delay disengagement when alternatives are limited (Bu et al., 2025). Thus, resilience could mediate the relationship between future work self clarity and decision-making difficulties.

H2: Resilience mediates the relationship between future work self clarity and career decision-making difficulties.

In contrast, self-efficacy is widely regarded as a protective resource in career decision-making, enhancing perceived control and reducing anxiety. Empirical research shows that self-efficacy positively predicts career adaptability and reduces decisional strain (Söner and Yılmaz, 2025). Accordingly, we expect self-efficacy to mediate the effects of future work self clarity on career decision-making difficulties.

H3: General self-efficacy mediates the relationship between future work self clarity and career decision-making difficulties.

Finally, psychological resource models suggest that resilience and self-efficacy may operate simultaneously through competing pathways, with resilience sustaining engagement and self-efficacy facilitating decisional confidence (Bernabé et al., 2023). These resources may jointly transmit the effects of future work self clarity on career decision-making difficulties.

H4: Resilience and general self-efficacy jointly constitute a multiple mediation pathway linking future work self clarity to career decision-making difficulties.

**Figure 1:**
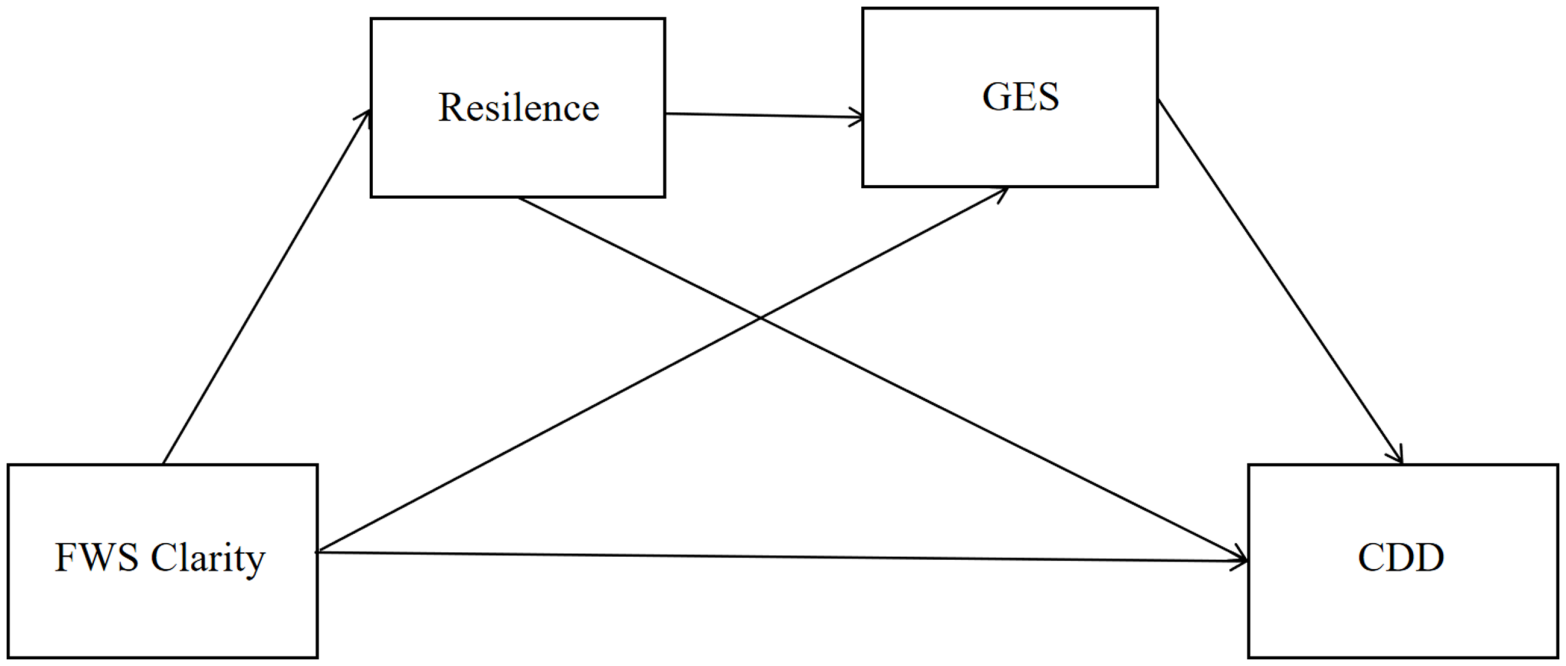
The theoretical model of multiple mediators.

From a resource-based and career construction perspective, career decision-making is shaped not only by the availability of psychological resources, but also by how different resources are activated and function under conditions of uncertainty. Although future work self clarity has been widely conceptualized as a motivational and self-regulatory asset, its benefits may not unfold uniformly across individuals. In particular, resilience and general self-efficacy—two psychological resources commonly regarded as adaptive—may play distinct roles in the translation of future-oriented cognitions into career decision-making outcomes. However, existing research has rarely examined how these resources operate simultaneously within a unified framework. Therefore, the present study seeks to examine the joint roles of resilience and general self-efficacy in the association between future work self clarity and multidimensional career decision-making difficulties among Chinese university students (see Figure 1).

## 2 Method

### 2.1 Participants and procedure

Participants were recruited using convenience sampling from several universities located in urban regions of China. Initially, 1,600 undergraduate students agreed to participate and completed the self-report questionnaire. After excluding 111 questionnaires with substantial missing data or invalid response patterns, the final analytic sample consisted of 1,498 participants, yielding an effective response rate of 90.78%.

Several data-screening criteria were applied to ensure response quality. First, respondents whose completion time was less than one-third of the sample’s average response time were excluded to reduce the influence of rapid or inattentive responding. Second, questionnaires that failed two embedded attention-check items were removed. In addition, cases displaying uniform response patterns (e.g., long strings of identical responses across items) were excluded to minimize mechanical responding.

The final sample comprised 57.14% female and 42.86% male students. Participants were distributed across academic years, including first-year (35.48%), second-year (28.22%), third-year (19.62%), and fourth-year students (16.68%). The study protocol was approved by the Institutional Review Board (IRB) of the authors’ affiliated institution. All participants provided written informed consent prior to data collection and were informed of the voluntary and anonymous nature of their participation.

### 2.2 Measures

#### 2.2.1 Future work self clarity

Future work self clarity was assessed using the Future Work Self Salience/Clarity Scale developed by Strauss et al. (2012) and subsequently validated and adapted for the Chinese cultural context by Guan et al. (2014). The scale consists of four items measuring a single dimension of clarity regarding one’s future work self. Responses were recorded on a 5-point Likert scale ranging from 1 (strongly disagree) to 5 (strongly agree).

This measure has demonstrated strong structural validity and cross-contextual stability across multiple cultural settings (Strauss et al., 2012; Guan et al., 2014). In the present sample, the scale exhibited excellent internal consistency (Cronbach’s *α* = 0.918), exceeding recommended psychometric standards (Nunnally and Bernstein, 1978).

#### 2.2.2 Resilience

Resilience was measured using the Brief Resilience Scale (BRS), which assesses individuals’ capacity to recover from stress. The Chinese version revised and validated by Chen et al. (2020) was used in this study. The BRS comprises six items rated on a 5-point Likert scale, with three items reverse-coded.

The BRS has been widely employed in international research and has demonstrated robust psychometric properties and cross-cultural applicability (Smith et al., 2008). In the present study, the scale showed excellent internal consistency reliability (Cronbach’s *α* = 0.932), meeting established reliability criteria (Nunnally and Bernstein, 1978).

#### 2.2.3 General self-efficacy

General self-efficacy was assessed using the General Self-Efficacy Scale (GSE; Schwarzer and Jerusalem, 1995), employing the Chinese version adapted by Wang et al. (2001). The scale consists of 10 items measuring a single latent dimension of perceived competence in coping with challenges and accomplishing tasks. Responses were rated on a 4-point Likert scale.

The GSE has been extensively validated across diverse cultural contexts and has demonstrated sound psychometric properties in Chinese samples (Wang et al., 2001). In the current study, the scale demonstrated good internal consistency (Cronbach’s *α* = 0.867), consistent with recommended standards (Nunnally and Bernstein, 1978).

#### 2.2.4 Career decision-making difficulties

Career Decision-Making Difficulties were measured using the Chinese version of the Career Decision-Making Difficulties Questionnaire (CDDQ), revised by Li (2009) based on the questionnaire originally translated and adapted by Li Xiying. The scale was refined through exploratory and confirmatory factor analyses and consists of 35 items assessing three core dimensions: lack of readiness, difficulties in information exploration, and decisional conflicts.

Responses were recorded on a 5-point Likert scale. The theoretical framework of this instrument is grounded in the internationally established CDDQ developed by Gati et al. (1996) and further elaborated by Gati and Levin (2014). In the present sample, the overall scale demonstrated excellent internal consistency (Cronbach’s α = 0.96), indicating strong reliability (Nunnally and Bernstein, 1978).

## 3 Data analysis

All statistical analyses were conducted using SPSS version 26.0. Descriptive statistics, including means, standard deviations, correlation coefficients, and reliability indices, were computed for all study variables. Prior to hypothesis testing, key model assumptions were evaluated. Linearity was examined through scatterplots, which indicated linear relationships between predictors and outcome variables.

Multicollinearity was assessed by calculating variance inflation factors (VIFs) for all predictors. All VIF values were well below the conservative threshold of 5, suggesting that multicollinearity was not a concern. Missing data were handled using maximum likelihood estimation, which is considered an efficient and unbiased approach under the assumption of data missing at random (MAR).

To examine the proposed multiple mediation model, the PROCESS macro (Model 6; Hayes, 2018) was employed. Consistent with recent methodological recommendations emphasizing theory-driven modeling, the analyses focused on testing the internal dynamics among the focal psychological constructs. To account for potential demographic confounding, gender (sex) and grade were included as covariates in all regression equations. This approach allowed for a more rigorous estimation of the proposed mediation pathways while ensuring that the observed effects were not attributable to differences in demographic background.

### 3.1 Descriptive statistics and correlational analyses

Descriptive statistics and zero-order correlations among all focal variables are presented in Table 1. Overall, participants exhibited moderate mean levels and acceptable variability in future work self clarity (FWSC; M = 3.41, SD = 0.65), resilience (M = 2.78, SD = 0.36), general self-efficacy (GSE; M = 3.24, SD = 0.72), and career decision-making difficulties (CDD; M = 2.91, SD = 0.55).

**Table 1:** Descriptive statistics of variables and their correlations.

Variable	*M*	SD	1	2	3	4
FWCS	3.41	0.65	-			
Resilience	2.78	0.36	0.190^***^	-		
GES	3.24	0.72	0.414^***^	0.153^***^	-	
CDD	2.91	0.55	−0.118^***^	0.403^***^	−0.201^***^	-

^*^p < 0.05, ^**^p < 0.01, ^***^p < 0.001.

Correlational analyses revealed meaningful associations among the study variables. FWSC was positively correlated with resilience (r = 0.190, *p* < 0.001) and GSE (r = 0.414, *p* < 0.001), and negatively correlated with CDD (r = −0.118, *p* < 0.001). Resilience was positively associated with GSE (r = 0.153, *p* < 0.001). In addition, GSE was negatively correlated with CDD (r = −0.201, *p* < 0.001). Notably, resilience showed a significant positive correlation with CDD (r = 0.403, *p* < 0.001), suggesting a potentially complex role of resilience in the career decision-making process.

### 3.2 Tests of the multiple mediation model

Consistent with prior research demonstrating the conceptual equivalence between regression-based mediation analysis and SEM-based indirect effect estimation, Hayes’ PROCESS macro was employed to test the hypothesized multiple mediation model. Specifically, Model 6 was estimated with 5,000 bootstrap resamples to examine the mediating roles of resilience and general self-efficacy in the association between future work self clarity (FWSC) and career decision-making difficulties (CDD).

To account for potential demographic confounding, gender (sex) and grade were included as covariates in all regression equations. All coefficients reported below are unstandardized estimates.

Given the mixed empirical evidence regarding future-oriented cognitions under conditions of high uncertainty, and the possibility that future work self clarity may function as both a motivational resource and a source of cognitive pressure, a non-directional hypothesis was adopted for the direct association between FWSC and CDD.

The total effect of FWSC on CDD was significant and negative (*β* = −0.811, SE = 0.177, t = −4.585, *p* < 0.001), indicating that higher levels of future work self clarity were associated with fewer career decision-making difficulties. When resilience and general self-efficacy were simultaneously entered into the model, the direct effect of FWSC on CDD remained significant and negative (*β* = −0.775, SE = 0.172, t = −4.505, *p* < 0.001), suggesting partial mediation after controlling for gender and grade.

As shown in Table 2, FWSC significantly and positively predicted resilience (*β* = 0.313, *p* < 0.001). Among the covariates, gender was negatively associated with resilience (β = −0.913, *p* < 0.001), whereas grade showed a small but significant positive association with resilience (β = 0.265, *p* = 0.009). FWSC also significantly and positively predicted general self-efficacy (β = 0.552, *p* < 0.001). In addition, resilience was a significant positive predictor of general self-efficacy (*β* = 0.061, *p* = 0.002). Neither gender nor grade showed a significant association with general self-efficacy.

**Table 2:** Path coefficients of the multiple mediation model (non-standardized).

Predictor variable	Outcome variable	*β*	Boot SE	*t*	*p*	Boot LLCI	Boot ULCI
FWSC(X)	Resilience(M1)	0.313	0.042	7.491	<0.001	0.232	0.396
Sex	Resilience(M1)	−0.913	0.223	−4.098	<0.001	−1.350	−0.476
Grade	Resilience(M1)	0.265	0.101	2.620	0.009	0.067	0.464
FWSC(X)	GES(M2)	0.552	0.033	16.735	<0.001	0.487	0.616
Resilience(M1)	GES(M2)	0.061	0.020	3.055	0.002	0.022	0.101
Sex	GES(M2)	−0.142	0.174	−0.813	0.417	−0.483	0.200
Grade	GES(M2)	0.068	0.079	0.858	0.391	−0.087	0.222
FWSC(X)	CDD(Y)(c´)	−0.775	0.172	−4.505	<0.001	−1.113	−0.438
Resilience(M1)	CDD(Y)(b1)	1.929	0.097	19.976	<0.001	1.740	2.119
GES(M2)	CDD(Y)(b2)	−1.118	0.124	−9.028	<0.001	−1.361	−0.875
Sex	CDD(Y)	1.145	0.834	1.374	0.170	−0.490	2.781
Grade	CDD(Y)	−0.345	0.377	−0.913	0.361	−1.085	0.3

When predicting CDD, the two mediators exhibited effects in opposite directions. Resilience was a significant positive predictor of CDD (β = 1.929, *p* < 0.001), whereas general self-efficacy was a significant negative predictor of CDD (β = −1.118, *p* < 0.001). Neither gender nor grade significantly predicted CDD, indicating that the observed mediation effects were not attributable to these demographic factors.

Bootstrap analyses of indirect effects are presented in Table 3. The total indirect effect of FWSC on CDD was not statistically significant (β = −0.036, Boot SE = 0.140, 95% CI [−0.310, 0.240]). However, two specific indirect effects were statistically significant and operated in opposite directions. Specifically, the indirect effect of FWSC on CDD through resilience was positive and significant (Indirect Effect 1: β = 0.603, 95% CI [0.408, 0.809]), whereas the indirect effect through general self-efficacy was negative and significant (Indirect Effect 2: β = −0.617, 95% CI [−0.801, −0.437]).

**Table 3:** FWSC’s bootstrap analysis of CDD’s indirect effects.

Ind effect	Path	*β*	Boot SE	Boot LLCI	Boot ULCI
Ind1	X → M1 → Y	0.603	0.104	0.408	0.809
Ind2	X → M2 → Y	−0.617	0.092	−0.801	−0.437
Ind3	X → M1 → M2 → Y	−0.021	0.009	−0.042	−0.005
Total Ind	TOTAL	−0.036	0.140	−0.310	0.240

Because these two indirect effects were comparable in magnitude but opposite in direction, they offset each other, resulting in a nonsignificant total indirect effect. This pattern provides clear evidence for a competitive multiple mediation structure, even after controlling for demographic covariates.

**Figure 2:**
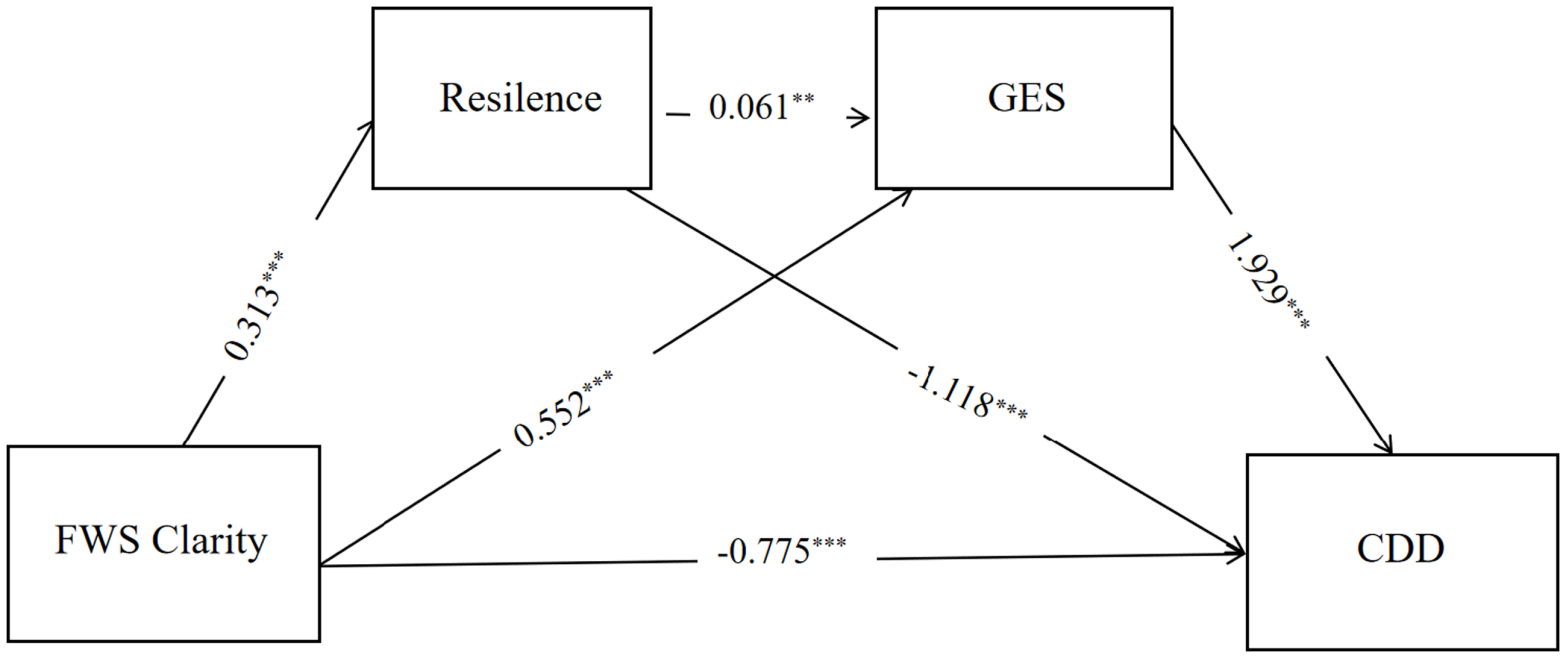
Multiple mediation model. Values represent unstandardized path coefficients controlling for sex. ^*^p < 0.05, ^**^p < 0.01, ^***^p < 0.001.

Finally, the serial indirect effect from FWSC to CDD through resilience and general self-efficacy (FWSC → Resilience → GSE → CDD) was also significant and negative (β = −0.021, 95% CI [−0.042, −0.005]) (see Figure 2).

## 4 Discussion

Grounded in an integrative perspective of future-oriented cognition and psychological resources, the present study systematically examined the multiple pathways through which future work self clarity (FWSC) influences career decision-making difficulties (CDD) via resilience and general self-efficacy (GSE) among university students. Overall, the findings demonstrate that the effect of FWSC on CDD does not follow a single, linear transmission route. Instead, FWSC operates through distinct types of psychological resources whose effects may compete with, offset, or even counteract one another. This pattern offers important theoretical and practical insights for contemporary career development research. Taken together, the present findings suggest that FWSC exerts its influence on career decision-making not through a uniformly facilitative mechanism, but through a dynamically balanced system of psychological resources with distinct regulatory functions. Importantly, these findings should be interpreted within the broader sociostructural context in which contemporary Chinese university students make career decisions. In recent years, intensified labor market competition, credential inflation, and narrowing opportunity structures have fundamentally altered the nature of career choice, shifting it from a problem of open-ended exploration to one of constrained selection under structural uncertainty. Within this context, psychological resources may function in qualitatively different—and sometimes counterintuitive—ways compared to resource—rich or opportunity-abundant settings. Importantly, all key effects were observed after controlling for gender and grade, indicating that the identified competitive mediation structure cannot be attributed to demographic differences in career stage or social positioning. Although gender and grade showed associations with resilience, they did not directly predict career decision-making difficulties, suggesting that demographic factors may shape the availability of certain psychological resources without determining decisional outcomes. The persistence of the core pathways after adjustment underscores the robustness of the proposed resource-based mechanism beyond background characteristics.

First, the direct negative association between FWSC and CDD confirms the foundational role of FWSC as a higher-order, proactive cognitive representation in career decision-making. FWSC does not reflect evaluative commitment or decisional closure; rather, it captures the cognitive accessibility and structural coherence of a future work identity. A clear future work self provides individuals with a cognitive anchor for filtering information and maintaining goal coherence, thereby reducing hesitation and value conflict during the decision process (Strauss et al., 2012; Voigt and Strauss, 2024). Importantly, however, the present results indicate that the benefits of FWSC do not automatically translate into lower levels of CDD. Rather, the psychological consequences of FWSC depend on the type and configuration of internal resources through which it is enacted, giving rise to qualitatively different cognitive and emotional processing pathways (Liu et al., 2023; Gürbüz et al., 2024).

Second, the findings concerning resilience represent a notable theoretical advancement. Although resilience has traditionally been conceptualized as a protective resource that buffers individuals against adversity (Jeamjitvibool et al., 2022), the present study reveals that resilience can function as a positive mediator between FWSC and CDD under certain conditions. This pattern suggests a paradoxical or “double-edged” role of resilience in career decision-making contexts (Liu et al., 2024). When future goals are highly salient and clearly defined, individuals high in resilience may exhibit heightened persistence and sustained engagement. While such persistence can be adaptive in resource-rich environments, it may become maladaptive under conditions of informational ambiguity or structural constraint, leading to excessive cognitive processing, emotional exhaustion, and heightened sensitivity to negative feedback (Troy et al., 2023; Gong et al., 2023). These findings resonate with prior work on goal–resource misfit and the adaptive costs of overpersistence (Bonanno et al., 2015; Liu et al., 2024). These findings therefore call for greater conceptual precision in how resilience is theorized and operationalized in career decision contexts, rather than being interpreted as a methodological artifact.

In contrast, general self-efficacy exhibited a stable and consistently protective function in the present study. GSE significantly reduced CDD by strengthening individuals’ sense of agency and confidence in their capacity to initiate and sustain goal-directed action (Lent and Brown, 2020, Xu, 2023; Levin et al., 2024). This robust inhibitory effect supports social cognitive career theory, which positions self-efficacy as a core mechanism underlying career exploration and the reduction of avoidance-based decision behaviors (Lent et al., 2002; Lent and Brown, 2020, Xu, 2023). From a process perspective, GSE appears to facilitate the translation of FWSC from an abstract future-oriented cognition into concrete strategies and executable plans, thereby mitigating emotional overload and decisional paralysis induced by information complexity or value conflict (Kwon, 2019; Zhou et al., 2024). In this sense, general self-efficacy differs fundamentally from resilience in its regulatory function: whereas resilience sustains engagement under adversity, self-efficacy directly supports decisional closure through action initiation and commitment. To further integrate these differentiated mediation patterns at a system level, the following section elaborates on the functional differentiation and competitive dynamics among psychological resource pathways.

### 4.1 Functional differentiation and competing psychological resource pathways

Beyond the examination of individual mediating pathways, the present findings point to a more nuanced configuration of psychological resource processes activated by future work self clarity. Rather than exerting a uniformly facilitative influence through internal resources, FWSC appears to mobilize multiple regulatory mechanisms that operate in functionally differentiated—and at times countervailing—directions, consistent with broader models of personal resource dynamics that emphasize interactive resource processes rather than simple additive effects (Hobfoll, 1989; Halbesleben et al., 2014). From the perspective of conservation of resources (COR) theory, individuals strive to maintain, protect, and build valued resources, and the relative balance among different types of resources shapes stress and coping outcomes in complex ways (Hobfoll, 2001). When persistence-oriented resources such as resilience are activated alongside action-oriented resources such as general self-efficacy, their opposing influences may approximate a functional equilibrium, yielding minimal net change in aggregate decisional difficulty even as meaningful individual pathways remain active.

This pattern underscores a critical limitation of treating psychological resources as additive or uniformly adaptive. Aggregated indicators of “overall” psychological mediation may obscure theoretically meaningful processes that unfold at the level of specific resource functions, echoing findings that resilience and self-efficacy contribute differentially to stress within a conservation of resources framework (Halbesleben et al., 2014). In the present study, resilience and general self-efficacy do not simply differ in strength but in regulatory logic: the former sustains engagement under adversity, whereas the latter facilitates agentic resolution and behavioral commitment. The observed configuration thus reflects a functional counterbalance within the resource system, rather than the absence of mediation in a substantive psychological sense.

Importantly, the present study does not suggest that the individual indirect pathways should be interpreted in isolation or overemphasized independently of their combined, net effect. Such functionally differentiated and competing resource processes are likely to be especially salient in contexts characterized by high structural uncertainty and constrained opportunity structures. These findings should be interpreted within the sociocultural context of Chinese university students, whose career decision-making unfolds in a highly competitive and structurally constrained labor market. Under such conditions, persistence-oriented resources may sustain prolonged engagement with uncertain or suboptimal career options, potentially intensifying decisional strain when actionable opportunities remain limited, whereas efficacy-based resources are more likely to facilitate agentic resolution by enhancing confidence in initiating and regulating goal-directed behavior. Accordingly, career interventions should prioritize resource–task matching rather than the uniform enhancement of all positive psychological resources. Programs designed to strengthen future work self clarity may be most effective when coupled with efforts to cultivate actionable efficacy beliefs and realistic feasibility assessments, while resilience-building initiatives should be implemented cautiously to avoid inadvertently sustaining indecision under conditions of structural constraint (Arbona et al., 2024; Priyashantha, 2023; Liu et al., 2023).

From an integrative perspective, the present findings underscore that future work self clarity operates within a dynamically organized system of psychological resources whose effects are contingent on functional orientation and contextual constraints rather than additive strength. Recognizing this complexity is essential for interpreting the results appropriately and for delineating the conceptual boundaries of the present findings, thereby underscoring the importance of critically examining the methodological and measurement constraints that shape their interpretation and generalizability.

### 4.2 Implications for career development policy, education, and practice

Against the backdrop of increasing employment uncertainty and a growing policy emphasis on individual adaptability and resilience, the present findings offer timely insights into how future-oriented cognition and psychological resources should be differentially cultivated rather than uniformly promoted. By demonstrating that future work self clarity operates through functionally differentiated—and at times competing—psychological resource pathways, the study challenges one-size-fits-all approaches to career development and human capital interventions.

In educational contexts, particularly within higher education, the results suggest that enhancing future work self clarity alone is insufficient for reducing career decision-making difficulties. Career education programs that overemphasize persistence or psychological toughness may inadvertently intensify decisional strain when students face structurally constrained or ambiguous choice environments. In contrast, integrating future-oriented self-concept interventions with efficacy-building components—such as action planning, self-regulatory skills, and confidence in decision execution—may better facilitate adaptive career decision-making. These findings underscore the importance of a resource–task matching principle, whereby educational interventions are aligned with the specific decisional demands students encounter.

From a policy perspective, the findings caution against the uniform promotion of resilience as a universally adaptive trait in youth employment and entrepreneurship initiatives. While resilience may sustain engagement under uncertainty, it may also prolong indecision when feasible opportunities remain limited. Policymakers may therefore benefit from combining future-oriented motivational interventions with mechanisms that enhance decisional agency and feasibility appraisal, including structured career guidance, transparent labor market information, and opportunity signaling systems that support timely commitment rather than prolonged endurance of ambiguity.

The results also hold implications for organizational and entrepreneurial contexts. Although future-oriented self-concepts are often viewed as drivers of opportunity recognition and entrepreneurial intention, the present findings suggest that future clarity must be accompanied by sufficient efficacy beliefs to translate vision into action. Entrepreneurship support programs and early-career development initiatives may therefore be more effective when vision-oriented training is complemented by capability-building and decision execution support, rather than focusing exclusively on perseverance or passion.

Together, these applied implications highlight that the adaptive value of future work self clarity depends not on the accumulation of positive psychological resources, but on their functional configuration within specific institutional and decision contexts—an insight that underscores the importance of aligning career development interventions with the functional demands of specific decision contexts.

### 4.3 Practical implementation of resource–task matching: from conceptual principle to evidence-informed intervention

In response to increasing policy emphasis on adaptability, resilience, and future-oriented agency, the present findings offer concise implications for policy makers, regulatory and educational institutions, and organizations operating under labor market uncertainty. Rather than supporting the uniform enhancement of positive psychological resources, the results indicate that future work self clarity (FWSC) activates functionally differentiated resources whose effects on career decision-making may diverge or counteract one another, underscoring the importance of a resource–task matching perspective.

At the policy level, the findings caution against one-size-fits-all promotion of resilience as a universally adaptive trait. While resilience is frequently emphasized in employability and entrepreneurship initiatives, persistence-oriented resources may, under structurally constrained conditions, sustain prolonged engagement with ambiguous options and thereby intensify decision-making difficulties. Policies may therefore benefit from complementing resilience-oriented narratives with strategies that strengthen decisional agency and action execution (Savickas et al., 2019; Wang D. et al., 2024; Wang et al., 2024a, 2024b).

For regulatory and educational institutions, particularly in higher education, the results suggest that enhancing future-oriented self-concepts alone may be insufficient. Career guidance systems should prioritize feasibility appraisal and efficacy-based decision support rather than perseverance alone, aligning psychological resources with the functional demands of different decision stages (Fris et al., 2025; Wang D. et al., 2024; Wang et al., 2024a, 2024b).

At the organizational and entrepreneurial level, the findings indicate that future-oriented vision and persistence should be paired with efficacy-based support to facilitate timely decision execution. Without sufficient efficacy beliefs, vision-driven persistence may inadvertently prolong indecision under uncertainty.

Overall, these implications suggest that the adaptive value of future work self clarity lies not in the accumulation of positive psychological resources, but in their functional configuration within specific institutional and decision contexts.

## 5 Limitations and future directions

Several limitations of the present study warrant consideration. At the same time, clarifying these boundaries helps delineate the specific contributions of the present findings and provides concrete directions for future research.

First, the cross-sectional design limits causal inference regarding the temporal ordering (i.e., temporal precedence) among future work self clarity, psychological resources, and career decision-making difficulties (Wang D. et al., 2024; Wang et al., 2024a, 2024b). Although the proposed pathways are theoretically grounded, longitudinal or multi-wave designs are needed to examine temporal precedence and to test the stability of the observed competitive mediation structure over time.

Second, an important limitation concerns the measurement of resilience. The Brief Resilience Scale (BRS) conceptualizes resilience as the capacity to recover from adversity rather than as persistent goal pursuit or rigidity. Accordingly, the positive indirect association between resilience and career decision-making difficulties should not be interpreted as evidence that resilience reflects maladaptive persistence. Instead, recovery-oriented resilience may sustain prolonged engagement under uncertainty, which could delay timely goal disengagement when feasible options are constrained (Wrosch et al., 2003). Future research would benefit from incorporating measures of goal disengagement, flexibility, or rigid persistence to more directly test the proposed mechanisms.

Third, the exclusive reliance on self-report data collected from a single source may raise concerns about common method variance. While the presence of opposing indirect effects suggests that such bias is unlikely to fully account for the findings, future studies should incorporate multi-source data, behavioral indicators, or experimental manipulations of future-oriented cognition to strengthen causal and construct validity.

Finally, the generalizability of the findings may be shaped by the sociocultural context of Chinese university students, whose career decision-making occurs under conditions of heightened competition and structural constraint. Future cross-cultural research is needed to determine whether the observed configuration of competing psychological resource pathways extends to other institutional and cultural settings.

## 6 Conclusion

Drawing on an integrative framework of future-oriented cognition and psychological resources, the present study elucidated the mechanisms through which FWSC, resilience, and GSE jointly shape career decision-making difficulties. The findings demonstrate that FWSC does not exert a unidirectional influence on career decision-making; instead, its psychological impact is contingent upon the nature of the resource pathways through which it is enacted.

Specifically, resilience emerged as a positive mediator between FWSC and CDD, revealing its paradoxical function under high-pressure conditions, whereas general self-efficacy functioned as a stable negative mediator and constituted the primary resource through which FWSC alleviated decision difficulties. The divergence in these pathways forms a competitive mediation structure, indicating that the effects of FWSC depend on which psychological resource dominates the individual’s internal system.

Moreover, the identified serial mediation pathway (FWSC → Resilience → GSE → CDD) highlights the compensatory and cross-system dynamics among psychological resources. Collectively, the proposed “resource differentiation–resource competition” framework advances understanding of the formation of career decision-making difficulties and offers a more nuanced theoretical basis for both future research and evidence-based career interventions.

## Data Availability

The raw data supporting the conclusions of this article will be made available by the authors, without undue reservation.
